# Universal mobile protection system for aerosol-generating medical interventions in COVID-19 patients

**DOI:** 10.1186/s13054-020-02969-5

**Published:** 2020-05-26

**Authors:** Florian Straube, Clemens Wendtner, Ellen Hoffmann, Munich Clinic Collaboration Group, S. Volz, U. Dorwarth, M. Engel, N. Schneider, J. Lärmer, B. Nagel, P. Friederich, R. Fisch, A. Riess, J. Benedikter, F. J. Meyer, B. Lewerenz, W. Schepp, M. Schmid, C. Dodt, W. Schmidt, K. Weidenbach, S. Rogowski, H. Kossmann, M. Berger, C. Gatos, B. Wuerstl, M. Deichstetter

**Affiliations:** 1 Department of Cardiology and Internal Intensive Care Medicine, Munich Clinic Bogenhausen, Academic Teaching Hospital, Technical University of Munich (TUM), Englschalkinger Str. 77, 81925 Munich, Germany; 2 Department of Cardiology, Pneumology and Internal Intensive Care Medicine, Munich Clinic Schwabing, Academic Teaching Hospital, Ludwig-Maximilians-University (LMU), Munich, Germany; 3 Department of Hematology, Oncology, Immunology, Palliative Medicine, Infectious Diseases and Tropical Medicine, Munich Clinic Schwabing, Academic Teaching Hospital (LMU), Kölner Platz 1, 80804 Munich, Germany

**Keywords:** Aerosol-generation medical interventions, Healthcare personnel, High-flow nasal cannula oxygen, Non-invasive ventilation, COVID-19, SARS-CoV-2, Virus transmission, Protection gear, Shield

Refers to:

Huang L, Lin G, Tang L, Yu L, Zhou Z. Special attention to nurses’ protection during the COVID-19 epidemic. Crit Care. 2020;24(1):120.

SARS-CoV-2 can actively replicate in the upper respiratory tract and is shed for a prolonged time after symptoms end [1]. The prolonged viral shedding in sputum is relevant for hospital infection control [1]. Hospital-related transmission of the virus is a large threat to healthcare workers [2] especially if COVID-19 patients are treated by non-invasive ventilation or high-flow nasal oxygen [3].

Leonard et al. have recently proposed to use a surgical mask for the patient treated by high-flow nasal oxygen. At 40 L × min^−1^, the surgical mask captured 83.2% of particles [3]. It remains unclear if this is effective with increased flow velocities, and it does not apply to many aerosol-generating medical interventions.

For healthcare workers performing aerosol-generating procedures on patients with COVID-19, using fitted respirator masks (e.g., N95 respirators) in addition to other personal protective equipment (i.e., gloves, gown, eye protection, such as a face shield or goggles) has been recommended [4]. This equipment is mainly based on disposable materials, and the supply is limited in the context of the pandemic [5].

A new mobile and reusable protection system has been established. Medical staff might use it in addition to the personal protection measures already in operation.

The construction (Fig. 1) is made of a commercially available and easy to process opaque aluminum composite panel (bottom) on swivel castors and a transparent acrylic glass (top). A detailed description is available (DOI 10.31219/osf.io/2s93d; https://osf.io/2s93d/).

**Fig. 1:**
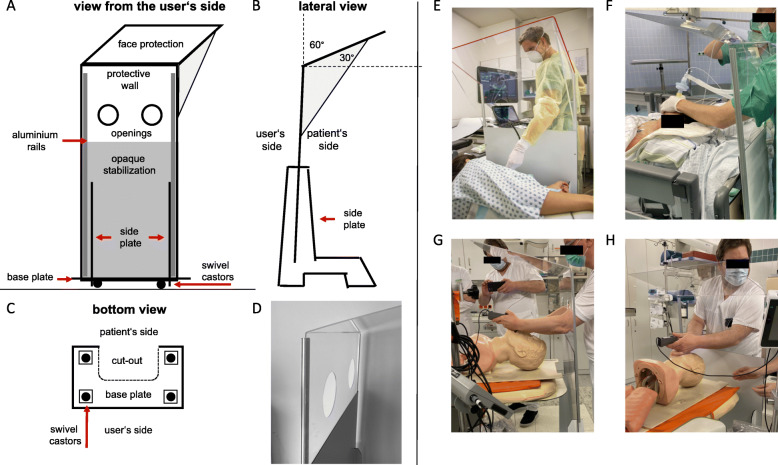
The universal mobile protection system. Schematic outline from **a** the user’s side, **b** the lateral view, and **c** the bottom view. **d** Picture of the top of the system, view is towards the patient’s side, and two openings are in the middle of shield. **e** Protection shield used during transesophageal echocardiography. **f** Picture taken in the theater just after extubation of a patient. **h** The prototype was used in a test track: videolaryngoscopically guided rapid sequence intubation was trained with a dummy in combination with the protection system

Unique features of the system are as follows: protective equipment neither worn by staff nor patients, but is placed on the ground and can be moved around on castors; flexible system for confined spaces, in operating rooms or functional areas; the transparent protective screen with an angled field of vision; and side shields deflect and prevent aerosols to be inhaled by the user. Openings allow personnel to treat patients without significantly reducing the shielding effect. The shielding has been visualized by steam tests (videos are provided online https://osf.io/7u2tv).

It might be used in addition to established protection measures for aerosol-generating procedures, e.g., for patient care during high-flow or non-invasive ventilation therapy, in-/extubation, upper GI endoscopy, bronchoscopy, transesophageal echocardiography, or drainage.

In those times, disposable protection gear is scarce, and the robust, easy-to-disinfect, reusable, mobile protection system might be helpful for medical personnel to work more safely in vulnerable situations. The universal, mobile protection system was evaluated in a test track and is considered useful by the main medical disciplines involved in the treatment of COVID-19 patients.

## Data Availability

1. Detailed description of the construction of the protection system: DOI 10.31219/osf.io/2s93d; https://osf.io/2s93d 2. Steam test videos: protection system with and without side shields DOI 10.17605/OSF.IO/7U2TV, Open Access Download: https://osf.io/7u2tv 1 Protectionsystem no side shields _ lat view 2 Protectionsystem with side shields _ lat view 3 Protectionsystem no side shields _ frontal view 4 Protectionsystem with side shields _ frontal view 3. Information sheet in English and German language DOI 10.17605/OSF.IO/7U2TV Open Access Download: https://osf.io/7u2tv

## Abbreviations

COVID-19: Corona virus disease 2019
N95: A medical mask meeting the N95 National Institute for Occupational Safety and Health air filtration rating (USA)
SARS-CoV-2: Severe acute respiratory syndrome coronavirus 2

## References

[1] Woelfel RC, Corman VM, Guggemos W, Seilmaier M, Zange S, Mueller MA, Niemeyer D, Jones Kelly TC, Vollmar P, Rothe C, Hoelscher M, Bleicker T, Bruenick S, Schneider J, Ehmann R, Zwirglmaier K, Drosten C, Wendtner C. Virological assessment of hospitalized cases of coronavirus disease 2019. Nature. 2020. 10.1038/s41586-020-2196-x.

[2] Huang L, Lin G, Tang L, Yu L, Zhou Z (2020). Special attention to nurses’ protection during the COVID-19 epidemic. Crit Care.

[3] Leonard S, Atwood CW Jr, Walsh BK, et al. Preliminary findings of control of dispersion of aerosols and droplets during high velocity nasal insufflation therapy using a simple surgical mask: implications for high flow nasal cannula. Chest. 2020. 10.1016/j.chest.2020.03.043. 10.1016/j.chest.2020.03.043 PMC7130245 32247712

[4] Alhazzani Waleed, Møller Morten Hylander, Arabi Yaseen M., Loeb Mark, Gong Michelle Ng, Fan Eddy, Oczkowski Simon, Levy Mitchell M., Derde Lennie, Dzierba Amy, Du Bin, Aboodi Michael, Wunsch Hannah, Cecconi Maurizio, Koh Younsuck, Chertow Daniel S., Maitland Kathryn, Alshamsi Fayez, Belley-Cote Emilie, Greco Massimiliano, Laundy Matthew, Morgan Jill S., Kesecioglu Jozef, McGeer Allison, Mermel Leonard, Mammen Manoj J., Alexander Paul E., Arrington Amy, Centofanti John E., Citerio Giuseppe, Baw Bandar, Memish Ziad A., Hammond Naomi, Hayden Frederick G., Evans Laura, Rhodes Andrew (2020). Surviving Sepsis Campaign: guidelines on the management of critically ill adults with Coronavirus Disease 2019 (COVID-19). Intensive Care Medicine.

[5] Emanuel Ezekiel J., Persad Govind, Upshur Ross, Thome Beatriz, Parker Michael, Glickman Aaron, Zhang Cathy, Boyle Connor, Smith Maxwell, Phillips James P. (2020). Fair Allocation of Scarce Medical Resources in the Time of Covid-19. New England Journal of Medicine.

